# Analyzing Misinformation and Disinformation: Understanding Swiss COVID-19 Narratives Through Natural Language Processing Analysis

**DOI:** 10.2196/76441

**Published:** 2026-03-30

**Authors:** Federico Germani, Giovanni Spitale, Franc Fritschi, Sonja Merten, Nikola Biller-Andorno

**Affiliations:** 1 Institute of Biomedical Ethics and History of Medicine, University of Zurich, Winterthurerstrasse 30, Zürich, 8006, Switzerland, 41 44 634 40 80; 2 Swiss Tropical and Public Health Institute, University of Basel, Basel, Switzerland

**Keywords:** COVID-19, misinformation, disinformation, public discourse, natural language processing, Switzerland

## Abstract

**Background:**

The COVID-19 pandemic has highlighted the challenges posed by the rapid spread of misinformation and disinformation, exacerbating societal polarization and institutional distrust. Understanding how misinformation and disinformation is understood and framed in public discourse is essential to developing strategies for building societal resilience and promoting informed decision-making during crises.

**Objective:**

This study explores the use of the terms misinformation and disinformation across Swiss public discourse during the COVID-19 pandemic, examining their framing within newspaper articles and social media interactions. The findings aim to inform policymakers and journalists or communicators on mitigating the societal impact of misinformation and disinformation through the promotion of a common understanding of the terms misinformation and disinformation.

**Methods:**

We analyzed 2 datasets using a natural language processing pipeline, including lemmatization, co-occurrence analysis, and semantic network mapping: media articles retrieved via Factiva and social media posts collected via CrowdTangle.

**Results:**

The framing of misinformation and disinformation varied significantly across the datasets. News media highlighted its role in shaping public sentiment, often discussing the tension between journalistic integrity and the amplification of falsehoods. Social media exhibited polarized narratives, with discussions centered on conspiracy theories, distrust in institutions, and grassroots mobilization.

**Conclusions:**

Diverging narratives on the very concepts of misinformation and disinformation across public discourse reflect broader societal tensions. Robust journalistic integrity in the media and resilience strategies against misinformation and disinformation involving empowering publics through information literacy approaches are critical to bridging divides and reducing polarization.

## Introduction

The COVID-19 pandemic has not only presented unprecedented public health challenges but has also exposed the susceptibility of public discourse to the rapid dissemination of misinformation and disinformation [1]. This phenomenon—which includes intentional falsehoods and unintentional inaccuracies [2]—has amplified societal divisions [3], diminished trust in institutions [4], and complicated efforts to manage the crisis effectively [5]. Misinformation refers to inaccuracies shared unknowingly by people, thinking the information is accurate, whereas disinformation refers to falsehoods disseminated deliberately to deceive with intent to harm, usually for political gains or economic reasons [6]. Despite being distinct concepts, here, we discuss both, as the focus of this work is on their framing and impact on public discourse, and because this distinction might not always be clear to lay publics using the terms. Misinformation and disinformation have been implicated in undermining vaccine uptake [7], conspiracy theories [8], and eroding confidence in evidence-based measures [9].

As a fundamental element of societal resilience, a transparent and reasoned public discourse is essential during crises [10,11]—for instance, in Italy, public communication during the COVID-19 pandemic combined institutional messages with emotionally resonant media campaigns, highlighting collective responsibility and solidarity [12]. However, the fragility of such discourse is not only due to the circulating misinformation and disinformation but also due to the contentious and often polarized use of the terms “misinformation” and “disinformation” [13,14]. These terms have frequently been used in emotionally charged and politicized contexts, becoming subjects of debate themselves, rather than tools for clarity and common understanding [13-15]. Scholarly debates have abundantly explored how misinformation has been shaped and propagated during the COVID-19 pandemic [4,16,17], yet the specific framing of misinformation and disinformation in public discourse—whether in media narratives or on social media platforms—remains underexplored, especially in the Swiss context.

This paper contributes to addressing this gap by analyzing how misinformation and disinformation have been framed—referring to the process of selecting and emphasizing certain aspects of reality to promote specific interpretations, evaluations, or solutions to an issue [18]—in Swiss public discourse during the COVID-19 pandemic. Given the different languages and cultures coexisting in Switzerland, the country’s unique sociocultural and political system presents a unique case study for examining the interplay between national narratives and regional dynamics. Our findings aim to inform strategies for promoting societal resilience and a shared understanding of the concepts of misinformation and disinformation across diverse audiences. This study is part of the broader research project titled “Boosting Public Discourse: A Targeted Evidence-Based Strategy to Improve Moral Reasoning” [19], which examines the framing of key moral terms, such as *freedom* [20], in Switzerland during the COVID-19 pandemic. Here, we focus on misinformation and disinformation, analyzing its conceptualizations in Swiss public discourse as reflected in news media and social media during the COVID-19 pandemic.

## Methods

### Ethical Considerations

This study analyzed secondary data consisting exclusively of publicly available news articles and publicly accessible social media posts from Facebook pages and groups. No human participants were recruited or contacted, and no identifiable private or sensitive personal data were collected. The analysis was conducted on aggregated textual data, and no attempt was made to identify individual users. According to the Swiss Federal Act on Research involving Human Beings (Human Research Act), research that does not involve human participants, identifiable health-related data, or biological material falls outside the scope of the Act and does not require review by a cantonal ethics committee [21]. As this study relied solely on publicly accessible, nonidentifiable data, formal ethics approval was not required. All procedures complied with applicable data protection regulations and institutional standards for responsible research.

### Data Collection

The data used in this study include 2 comprehensive datasets, each curated to capture a wide range of public discourses surrounding the COVID-19 pandemic. The first dataset (news articles) consists of 209 articles sourced from the Dow Jones Factiva database [22], covering the period from September 2019 to April 2023. The query was specifically designed to extract articles discussing COVID-19, misinformation, and disinformation, focusing on media reporting on these topics in relation to Switzerland. It is important to note that the corpus does not consist solely of articles published by Swiss media. Due to the query logic in Factiva, which does not allow for country-level granularity in source definition, the dataset also includes articles about Switzerland that were published by foreign media outlets. Moreover, recognizing that Swiss audiences regularly consume foreign media, the query was designed to capture a diverse range of journalistic narratives. Consequently, the dataset encompasses news articles in German, French, Italian, and English that discuss Switzerland and issues related to misinformation and disinformation, thereby providing a more comprehensive insight into how these narratives were framed and disseminated in public discourse. The full details on the data collection, including the formulation of the different queries, are available in this study’s OSF repository [23]. The second dataset (social media posts) comprises 580 posts collected via CrowdTangle [24], focusing on public interactions on Facebook pages and groups. These posts, gathered between September 2019 and April 2023, were drawn from Swiss Facebook pages and groups where discussions of COVID-19–related topics occurred. The query targeted the keywords “misinformation” (in German: *Fehlinformation*) and “disinformation” (in German: *Desinformation*), capturing a spectrum of user-generated content, including opinions, debates, and reactions to public health measures. Here, geographical filtering was performed using CrowdTangle’s “local relevance” parameter, which limits the query to “posts from Pages or public groups that are predominantly local to that area” [25]. This analysis focuses primarily on German-language content. Further details on the data collection methodology and query formulation are available in the study’s OSF repository [23].

### Analysis

The analytical process in this study leverages natural language processing (NLP) tools and semantic network maps to extract meaningful insights from the data. The first step involved parsing and preprocessing the datasets to prepare them for analysis. For news articles, metadata, such as publication dates, sources, and article lengths, were extracted. As social media posts included some noise (posts not mentioning any of our COVID-19 descriptors), we filtered the corpus, removing posts not containing the following words: “covid,” “corona,” “virus,” “covid-19,” “coronavirus,” “pandemic,” “epidemic,” “outbreak,” “pandémie,” “épidémie,” “Pandemie,” “Epidemie,” “Seuche,” “pandemia,” and “epidemia.” Lemmatization was carried out using the NLP library spaCy to reduce words to their base forms [26]. This step enabled uniform analysis across variations of the same word (eg, “misinform,” “misinformation”). To capture and describe the semantic space of lemmas, we applied co-occurrence analysis, a method that examines the frequency and patterns of lemmas appearing together within a defined textual context—in our case, sentences. This approach allows us to identify semantic relationships between terms, uncovering underlying structures and associations in the data. Semantic network maps were generated using Gephi, an open-source software for network data analysis [27]. These maps provided a high-level perspective on the terms most frequently co-occurring with “misinformation” and “disinformation” across the datasets. In these maps, nodes represent terms, with their size proportional to frequency, while edges indicate the strength of co-occurrence between them. The topology of the maps, shaped by the Circle Pack, Noverlap, and Label Adjust layouts, reflects the organization of terms within thematic clusters, which were determined by modularity class [28]. Color-coding based on modularity class further emphasizes these clusters, providing a clear and intuitive visual representation of the relationships and structure within the discourse. The modularity class analysis was conducted with a resolution parameter set to 0.5, which determines the granularity of the clusters detected [29]. A resolution of 0.5 was chosen empirically and informed by prior research [30] to ensure meaningful thematic groupings. In the analysis, we considered the largest and most prominent thematic clusters of each dataset (see the Results section).

To complement the quantitative analysis, we manually selected narrative examples to contextualize the findings. These examples illustrate specific uses of the key terms “misinformation” and “disinformation” within distinct contexts. This approach adds depth by showcasing how these terms are used and perceived in real-world scenarios. NLP techniques can efficiently process large volumes of text data (as in the case of our corpora), enabling us to systematically identify recurring patterns and lemmas that might be overlooked in manual analyses [31-33]. By integrating NLP analyses with narrative examples, we combine the computational efficiency of automated analysis with the context provided by individual narratives. This approach enables a more nuanced understanding of phenomena and enhances the understandability and richness of research findings. Detailed methodological procedures and aspects are available in this study’s OSF repository [23].

## Results

### News Media (Factiva Dataset)

**Figure 1.**
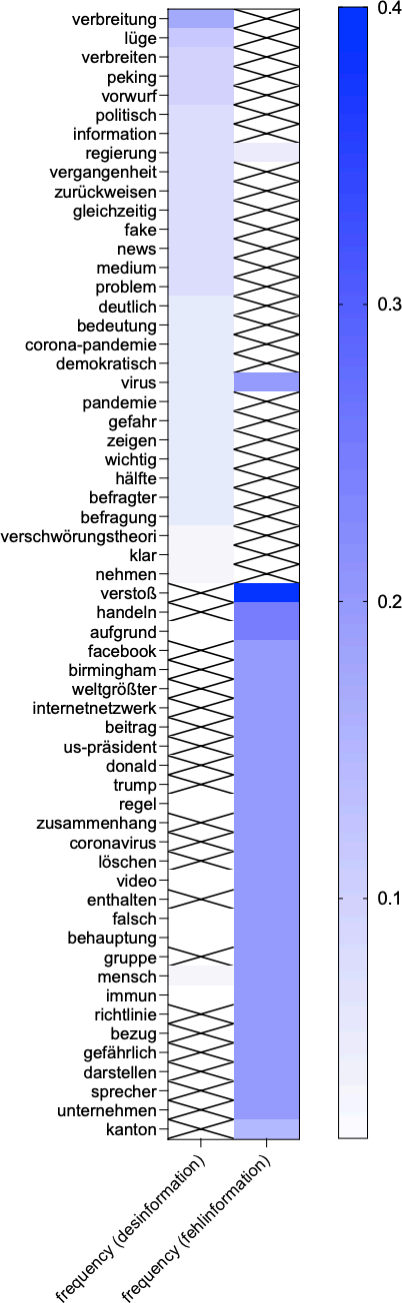
Co-occurrence plot of the terms most commonly associated with “misinformation” and ”disinformation” (*“Fehlinformation” and “Desinformation”*) in the Factiva dataset on news articles. The plot illustrates the strength of co-occurrence between lemmas, with darker blue colors indicating a higher frequency of co-occurrence. The frequency represents the number of times (in percentage, range 0‐1) in which a lemma has been found in association with “misinformation” and ”disinformation” (either “*Desinformation”* or “*Fehlinformation”*), meaning they were found in the same sentence. Crossed cells indicate no co-occurrence between the listed lemmas and the terms “misinformation” and ”disinformation” (either “*Desinformation”* or “*Fehlinformation”*).

**Figure 2.**
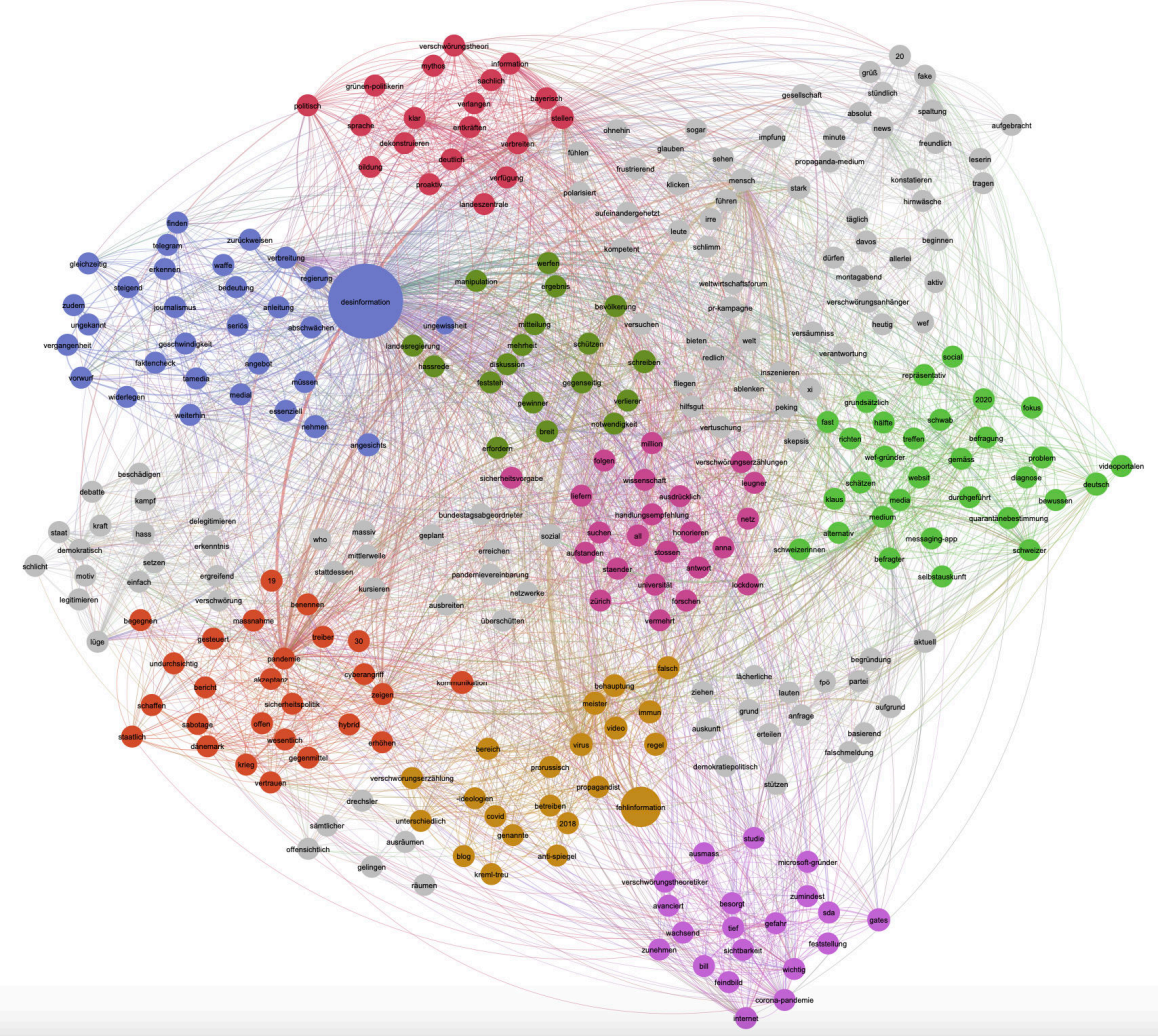
Semantic network map of the lemmas found in the dataset on news media articles (Factiva) associated with “misinformation” and ”disinformation.” Node size represents lemma frequency, edges represent co-occurrence, and color and topology represent modularity class.

The entire retrieved dataset included 209 articles. From the analysis of the news dataset obtained via Factiva, the NLP analysis visualized through the co-occurrence analysis plot of the terms most commonly associated with “misinformation” and “disinformation” (Figure 1) and the semantic network map of the terms co-occurring in association with “misinformation” and “disinformation” (Figure 2) reveals 5 relevant themes.

The first theme emerging from the co-occurrence analysis (Figure 1) and from the analysis of the semantic network map (Figure 2) is centered on addressing disinformation through critical engagement, education, and political discourse. “Verschwörungstheorie” (conspiracy theory) and “verbreiten” (to spread), both present also among the most co-occurring terms with “misinformation” and “disinformation” (with either “*Desinformation*” or “*Fehlinformation*”) (Figure 1), as well as the term “Mythos” (myth), point to narratives about the spread of false information and its role in fueling conspiracy theories, whereas “Bildung” (education), “entkräften” (to refute), “dekonstruieren” (to deconstruct), “proaktiv” (proactive), and “sachlich” (objective) indicate news media discussions about how to equip individuals with the tools to identify and counter misinformation and disinformation. These terms suggest a characterization of what traditional media consider misinformation and disinformation, complemented by a focus on promoting critical thinking and evidence-based discourse. In addition, the terms “klar” (clear) and “deutlich” (explicit), also among the most co-occurring terms with “misinformation” and “disinformation” (Figure 1), as well as the lemma “Sprache” (language), possibly reflect the importance of clear and transparent communication to provide information and counteract misinformation and disinformation effectively. Examples of articles’ excerpts discussing these topics include the following:

Die teils abstrusen Vorstellungen können reale Folgen haben. “Wir haben in der Forschung gesehen, dass Verschwörungstheorien zu einem stärkeren Misstrauen in Politik und in andere Menschen führten,” sagt Pummerer. “Verschwörungsanhänger haben auch weniger Vertrauen in andere Menschen und halten sich seltener an soziale Normen. Das hat Konsequenzen für den gesellschaftlichen Zusammenhalt.” / These sometimes abstruse ideas can have real consequences. “In our research, we have seen that conspiracy theories lead to greater mistrust in politics and in other people,” says Pummerer. “Conspiracy theorists also have less trust in other people and are less likely to adhere to social norms. This has consequences for social cohesion.”

[Row 14 Factiva dataset, November 19, 2021]

[…] Grundsätzlich kommt das Forschungszentrum zu dem Schluss, dass professionelle Medien durch die Pandemie nochmals an Bedeutung gewonnen haben, indem sie zuverlässige Orientierung bieten und die Desinformation limitieren. Eine «ungünstige Entwicklung» sei vor diesem Hintergrund, dass sich die ökonomische Krise des Journalismus nochmals akzentuiert habe, schreiben die Forscher. Während die Werbeeinnahmen im Print schon länger zurückgehen, waren sie erstmals auch im Online-Werbemarkt rückläufig. / The research center concludes that professional media have gained further importance during the pandemic by providing reliable information and limiting disinformation. Against this backdrop, the economic crisis in journalism has been further exacerbated, the researchers write. While print advertising revenues have been declining for some time, they have now also fallen for the first time in the online advertising market.

[Row 48 Factiva dataset, October 26, 2021]

Terms including “politisch” (political), among the most co-occurring terms with “misinformation” and “disinformation” (Figure 1), as well as the terms “Grünen-Politikerin” (Green Party politician) and “Landeszentrale” (state center), linked to the terms “misinformation” and “disinformation”, might suggest discussions about how political actors and institutions play a key role in shaping (the response to) misinformation and disinformation. Further, terms such as “Information” (relevant as one of the most common co-occurring terms with “misinformation” and “disinformation”), “Verfügung” (available), and “stellen” (to provide) within this cluster might highlight the need for readily accessible, reliable information, again, as a countermeasure to misinformation and disinformation.

Der immer wieder erhobene Vorwurf, Medien würden im Zusammenhang mit der Corona-Pandemie «Panikmache» betreiben, wird durch die Studie entkräftet. Während der ersten Welle, als die Situation neu und vieles noch unbekannt war, berichteten Journalistinnen und Journalisten in 16 Prozent der untersuchten Artikel alarmistisch über das Virus. Während der zweiten Welle traf das gemäss der Inhaltsanalyse des FÖG nur noch auf 6 Prozent der Artikel zu. Das heisst also: Ein Grossteil der fast 18’700 untersuchten Beiträge aus 60 Schweizer Medien war nicht alarmistisch.

The study refutes the repeatedly made accusation that the media engaged in ‘scaremongering’ in connection with the coronavirus pandemic. During the first wave, when the situation was new and much was still unknown, journalists reported alarmistically on the virus in 16 percent of the articles examined. During the second wave, according to the FÖG content analysis, this only applied to 6 percent of the articles. This means that a large proportion of the almost 18,700 articles examined from 60 Swiss media outlets were not alarmist.

[Row 30 Factiva dataset, October 25, 2021]

In general, this cluster highlights the interplay between misinformation and disinformation, political engagement, and education. It suggests that media portray the challenges of misinformation and disinformation as requiring action through educational initiatives and clear and transparent communication from institutions (and from the news media themselves). The terms point to a narrative in which facing the challenges posed by misinformation and disinformation involves empowering individuals through education and ensuring political, institutional, and journalistic accountability in providing factual information.

Gleichzeitig nimmt die Bedeutung von seriösem Journalismus angesichts der steigenden Verbreitung von Desinformation zu. Dies zeigt das am Montag veröffentlichte “Jahrbuch Qualität der Medien 2021” des Forschungszentrums Öffentlichkeit und Gesellschaft (Fög) der Universität Zürich. Abhilfe schaffen könnte allenfalls der Staat. “Es zeichnet sich immer mehr ab, dass qualitativ hochwertiger Journalismus nur durch eine direkte Medienförderung zu finanzieren ist,” wird Medien-Experte und Fög-Direktor Mark Eisenegger zitiert. / At the same time, the importance of serious journalism is increasing in the face of the spread of disinformation. This is shown in the “Jahrbuch Qualität der Medien 2021”(Yearbook of Quality of the Media 2021) published on Monday by the Research Center for the Public Sphere and Society (Fög) at the University of Zurich. The state could provide a remedy. “It is becoming increasingly apparent that high-quality journalism can only be financed through direct media funding,” said media expert and Fög director Mark Eisenegger.

[Row 8 Factiva dataset, October 25, 2021]

A second cluster highlighted in the semantic network map (Figure 2) suggests a theme focused on the difficulties of recognizing, verifying, and mitigating misinformation and disinformation, particularly within the context of rapid media dissemination and institutional credibility. In this context, the terms “Regierung” (government), “Vorwurf” (accusation), and “Verbreitung” (spread), all 3 among the most common co-occurring terms with “misinformation” and “disinformation,” as well as the term “Waffe” (weapon), might indicate disinformation being framed as a strategic tool that undermines governance and public trust. The terms suggest narratives involving accusations against governments and institutions, highlighting the adversarial use of disinformation in political contexts.

Sowohl die Schweiz als auch Österreich haben mit der SVP und der FPÖ starke rechte Parteien, die den Corona-Kurs der Regierung torpedieren.

In Österreich leben wir ja schon sehr lange mit der FPÖ, ebenso wie die Schweiz mit der SVP. Und es sollte eigentlich niemanden mehr verwundern, wie die beiden Parteien agieren. Aber in einer Krisensituation werden solche Kräfte nochmals zu einer besonderen Belastung. Wenn die Gesundheitssprecherin der FPÖ, die notabene eine Ärztin ist, sich hinstellt und wider alle Tatsachen behauptet, dass die Intensivstationen voller Opfer von Impfschäden seien, ist man schon mittendrin in der Verschwörungs- und Desinformationsszene. Nur sitzen diese Leute im Parlament.

Was macht das mit dem politischen Klima im Land?

Es schadet der Demokratie. Es geht dabei ja nicht mehr um unterschiedliche Meinungen. Es geht darum, dass politische Kräfte Desinformation und Lügen legitimieren. Wenn man das zulässt, lösen sich alle Parameter auf, nach denen Politik funktioniert. Dann ist alles erlaubt.

Both Switzerland and Austria have strong right-wing parties, the SVP and the FPÖ, that are undermining the government’s approach to the coronavirus.

In Austria, we have been living with the FPÖ for a very long time, as has Switzerland with the SVP. And no one should be surprised by the actions of either party. But in a crisis situation, such forces become a particular burden. When the FPÖ’s health spokeswoman, who is a doctor, stands up and claims, contrary to all facts, that the intensive care units are full of victims of vaccine damage, you are right in the middle of the conspiracy and disinformation scene. Only these people are in parliament.

What does that do to the political climate in the country?

It damages democracy. It is no longer about different opinions. It is about political forces legitimizing disinformation and lies. If you allow that to happen, all the parameters by which politics works are dissolved. Then anything goes.

[Row 39 Factiva dataset, December 23, 2021]

Furthermore, the terms “Journalismus” (journalism), “Faktencheck” (fact-checking), “medial” (media-related), and “Tamedia” (a Swiss media company) reflect discussions about the role of media as a channel for countering misinformation and disinformation.

Gemäss Selbstauskunft treffen Schweizerinnen und Schweizer besonders auf Social Media, Videoportalen und Messaging-Apps sowie Websites alternativer Medien auf Desinformation. Als Hauptquellen von Falschinformationen nehmen sie Aktivisten, Bürgerinnen, Politiker und Social Bots wahr. Grundsätzlich gelten eher Individuen als Urheber und weniger Organisationen.

According to the information they provided, the Swiss encounter disinformation particularly on social media, video portals and messaging apps, as well as on alternative media websites. They perceive activists, citizens, politicians and social bots as the main sources of misinformation. Individuals are generally considered to be the authors more often than organizations.

[Row 30 Factiva dataset, October 25, 2021]

Terms like “seriös” (serious) and “essenziell” (essential) within this cluster might point to the importance of reliable journalism in addressing the issue. The terms “Geschwindigkeit” (speed), “unbekannt” (unknown), and “Ungewissheit” (uncertainty) point to the challenges posed by the rapid and often untraceable spread of misinformation and disinformation, leading to heightened public uncertainty, and the term “zurückweisen” (reject), also among the most common co-occurring terms with “misinformation” and “disinformation”, as well as the terms “widerlegen” (refute) and “abschwächen” (weaken), reflect media narratives on the efforts to counter misinformation and disinformation through rebuttal and fact-checking. This cluster seems to highlight the systemic and persistent challenges posed by misinformation and disinformation, suggesting the existence within Swiss media discourse of a dual narrative: on one side, the rapid dissemination of misinformation and disinformation through social media exacerbates public uncertainty, weakening trust in institutions. On the other, it proposes that journalism plays a critical role in fact-checking and mitigating misinformation and disinformation.

The third cluster identified in the semantic network map (Figure 2) reflects a theme centered on the divisive nature of disinformation in public discourse and its role in shaping perceptions of governance and democracy. The terms “Hassrede” (hate speech) and “Lüge” (lie)—a term frequently linked with “misinformation” or “disinformation”—as well as the terms “Verlierer” (loser) and “gegenseitig” (mutual) suggest that misinformation and disinformation are part of a hostile and adversarial public discourse, especially in connection with referendums, possibly indicating that disinformation exacerbates societal divisions through polarizing narratives.

Die Reaktionen der siegreichen Befürworter des Covid-Gesetzes und jene der unterlegenen Gegner deuten nicht darauf hin, dass sich die Gräben zwischen den Lagern so rasch wieder schliessen lassen. Die Gewinner sprechen von einem Vertrauensbeweis aus Vernunft, die Verlierer von einer Irreführung.

So erwarten etwa die Grünen nach dem “pragmatischen und enorm wichtigen Ja” von den Gegnern des Gesetzes, dass diese das demokratische Ergebnis akzeptieren und “dazu beitragen, dass die Schweiz zu einer gesunden Diskussionskultur zurückfindet.” Auch die FDP hofft, dass “künftig wieder konstruktive Ansätze die Debatte prägen.”

Es sei zu hoffen, dass diejenigen, die sich als selbsternannte Freunde der Verfassung in Szene gesetzt hätten, sich “nun auch als Freunde der direkten Demokratie erweisen und das Abstimmungsergebnis akzeptieren,” hieben die Freidenker in die gleiche Kerbe. Sie hatten in Anlehnung an die gegnerischen “Freiheitstrychler” mit dem “Freiheitsimpfler” für ein Ja zum Gesetz geworben.

The reactions of the victorious proponents of the Covid law and those of the defeated opponents do not suggest that the rifts between the camps can be closed so quickly. The winners speak of a vote of confidence based on reason, the losers of a deception.

The Green Party, for example, expects the opponents of the law to accept the democratic result after the “pragmatic and enormously important yes” vote and to “help Switzerland to rediscover a healthy culture of discussion.” The FDP also hopes that “constructive approaches will shape the debate again in the future.”

It is to be hoped that those who had presented themselves as self-proclaimed friends of the constitution would now also prove to be friends of direct democracy and accept the result of the vote, the freethinkers took the same line. They had campaigned for a yes vote in favor of the law with the “Freiheitsimpfler” (freedom imposter), in reference to the opposing “Freiheitstrychler” (freedom truches).

[Row 47 Factiva dataset, November 11, 2021]

The presence within this cluster of the terms “Staat” (state) and “demokratisch” (democratic), also a relevant co-occurring term with “misinformation” and “disinformation” (Figure 1), highlights the interplay between misinformation and disinformation and perceptions of governance, suggesting that narratives within public discourse centered around disinformation undermine democratic principles and state legitimacy, as discussed before. Further, the terms “Manipulation” (manipulation), “legitimieren” (legitimize), and “Motiv” (motive) might reflect the intentional use of disinformation to influence public perception, delegitimize opponents, or justify certain actions. These terms point to the strategic framing of narratives to achieve specific political or social goals. The terms “Debatte” (debate), “Diskussion” (discussion), and “schreiben” (write) indicate that misinformation and disinformation often act as catalysts for contentious debates, suggesting that misinformation and disinformation drive public discourse but often in ways that deepen divisions rather than fostering understanding. Within this context, the terms “Gewinner” (winner), “Verlierer” (loser), and “Kraft” (force) appear to suggest that misinformation and disinformation further create competitive narratives where groups or ideas are framed as either victorious or defeated, which could lead to polarization and political or ideological struggles within the society. In sum, this cluster highlights how the media in Switzerland reported on the divisive and strategic nature of misinformation and disinformation in shaping public discourse and governance, reflecting a polarized environment where misinformation and disinformation fuels hostility and deepens societal divides while being used strategically to manipulate perceptions and outcomes. The terms in this cluster also point to the need for collective action to protect democracy.

A fourth cluster touches upon the dissemination of misinformation and disinformation through digital media channels, its ideological dimensions, and the resulting erosion of public trust. For example, the lemmas “prorussisch” (pro-Russian), “kreml-treu” (Kremlin-loyal), and “Propagandist” point to the role of propaganda and ideologically aligned media in spreading disinformation. These terms suggest a discourse and narrative concerning targeted disinformation campaigns originating from specific geopolitical actors or ideologies.

Zentrale Vorstellung ist, dass die Corona-Pandemie „seitens der Eliten seit Jahren vorbereitet wurde“ – eine gängige Darstellung von jenen, die ein weltweites konspiratives, selbstverständliches jüdisches Netz vermuten, das letztlich am Untergang des Abendlandes und an einer „Öko-Diktatur“ arbeitet. Und so mündet eine unsinnige Frage in die andere, immer davon ausgehend, dass die Pandemie von langer Hand – um nicht zu sagen von dunklen Mächten – vorbereitet wurde. Und da kommt dann die Frage – wie „diese Erkenntnisse bei Ihren Entscheidungen zu den Corona-Maßnahmen in der Zukunft“ berücksichtigt werden. Desinformation und Fake News sind schlicht und ergreifend keine Erkenntnisse, sie werden einfach aus den unterschiedlichsten Motiven in die Welt gesetzt – oft und sehr oft um demokratische Staaten zu delegitimieren.

The central idea is that the Corona pandemic “has been prepared by the elites for years”—a common belief of those who suspect a worldwide conspiratorial, self-evident Jewish network that is ultimately working on the downfall of the West and an “eco-dictatorship.” And so one nonsensical question leads to another, always assuming that the pandemic was prepared long in advance—not to mention by dark forces. And then comes the question of how “these findings will be taken into account in your decisions on corona measures in the future”. Disinformation and fake news are simply not insights; they are simply created for a wide variety of reasons—often and very often to delegitimize democratic states.

[Row 36 Factiva dataset, July 26, 2022]

The terms “Blog,” “Video,” “betreiben” (to operate), and “Bereich” (sector/field) within this cluster might underline the role of digital media platforms and blogs in amplifying misinformation and disinformation narratives. The terms “Covid,” “Virus,” “Immun” (immune), and “regel” (Rule), commonly co-occurring with the terms “misinformation” and “disinformation” (Figure 1), highlight that at least a part of the media discourse focused on health-related misinformation and disinformation and its societal implications. This cluster seems to be centered around discussions in Swiss media about the role of digital platforms and ideologically driven content, highlighting the intersection of conspiracy theories, health misinformation, and geopolitical propaganda, all contributing to a fragmented and polluted information landscape.

Finally, a fifth cluster touches upon themes around the influence of conspiracy theories, prominent individuals as targets of disinformation, and the evolving visibility of narratives in the context of the pandemic. For example, “Verschwörungstheoretiker” (conspiracy theorist), “Feindbild” (enemy image), and “Feststellung” (assertion) point to the centrality of conspiracy theories in misinformation and disinformation narratives. These terms suggest that conspiracy theorists shape the public’s perception by presenting simplified or distorted explanations of events, often targeting specific individuals or groups. Terms such as “Microsoft-Gründer” (Microsoft founder), “Bill,” and “Gates” indicate the focus on Bill Gates as a prominent figure in conspiracy theories related to COVID-19, where he is frequently portrayed as a central figure and used as an example of powerful individuals framed as scapegoats in disinformation narratives.

Der nicht erst in der Corona-Pandemie zum Feindbild von Verschwörungstheoretikern avancierte Microsoft-Gründer Bill Gates ist tief besorgt über die Verbreitung von Desinformation und Lügen im Internet. ‘Diese verrückten Ideen verbreiten sich irgendwie schneller in den sozialen Medien als die Wahrheit. Ich bin überrascht, dass mein Name in diesen Verschwörungstheorien auftaucht […]. Ich finde, dass es irgendwie ironisch ist, dass ich anmahnte, auf diese Pandemie vorbereitet zu sein - und jetzt gibt es Leute, die sagen, ich sei dafür verantwortlich.’ Der Multimilliardär appellierte an die Vernunft der Menschen: ‘Wir befinden uns inmitten einer Pandemie, und es ist wichtiger als je zuvor, sich mit den Tatsachen und der Wahrheit auseinanderzusetzen.’

Microsoft founder Bill Gates, who has been an enemy stereotype of conspiracy theorists since before the coronavirus pandemic, is deeply concerned about the spread of disinformation and lies on the internet. ‘Somehow these crazy ideas spread faster on social media than the truth. I’m surprised my name comes up in these conspiracy theories […]. I think it’s kind of ironic that I urged people to be prepared for this pandemic – and now there are people saying I’m responsible.’ The multibillionaire appealed to people’s common sense: ‘We are in the midst of a pandemic, and it’s more important than ever to deal with the facts and the truth.’

[Row 11 Factiva dataset, September 15, 2020]

And to be effective, conspiracy theories and disinformation are associated with an emotional manipulative intent: terms such as “besorgt” (worried) and “tief” (deep) reflect the emotional impact of disinformation, which can amplify fear and distrust among the public. Overall, this cluster highlights the presence, within Swiss media discourse, of a narrative focused on conspiracy theories and how they intersected with and shaped public perceptions and public discourse during the COVID-19 pandemic.

### Social Media (CrowdTangle Dataset)

**Figure 3.**
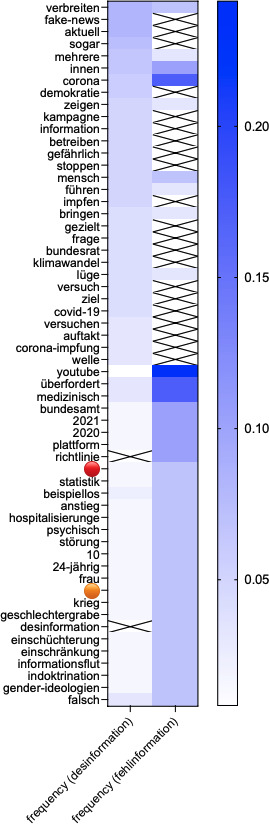
Co-occurrence plot of the terms most commonly associated with “misinformation” and ”disinformation” (*“Fehlinformation” and “Desinformation”*) in the CrowdTangle dataset on social media. The plot illustrates the strength of co-occurrence between lemmas, with darker blue colors indicating a higher frequency of co-occurrence. The frequency represents the number of times (in percentage, range 0‐1) in which a lemma has been found in association with “misinformation” and ”disinformation” (either “*Desinformation”* or “*Fehlinformation”*), meaning they were found in the same sentence. Crossed cells indicate no co-occurrence between the listed lemmas and the terms “misinformation” and ”disinformation” (either “*Desinformation”* or “*Fehlinformation”*).

**Figure 4.**
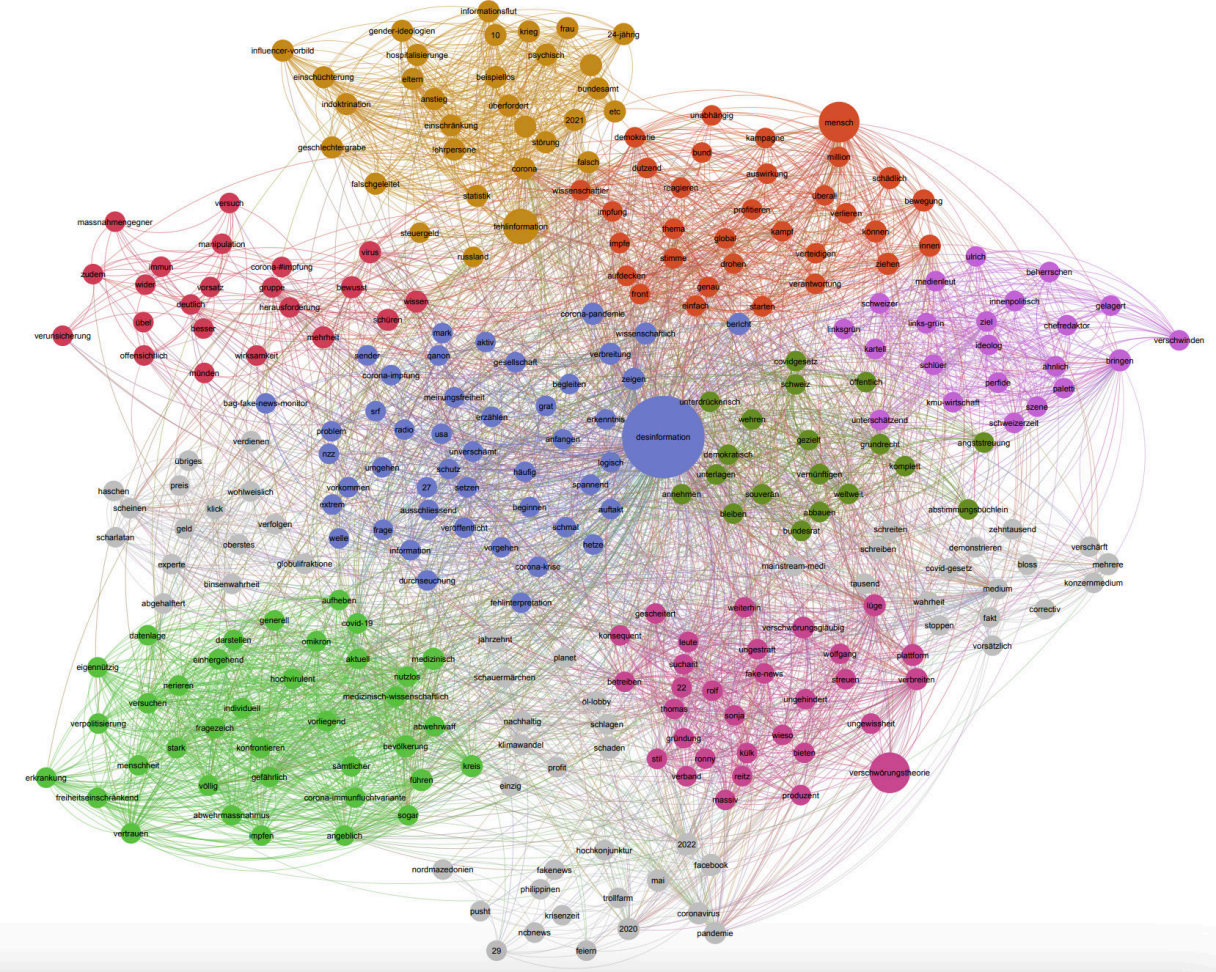
Semantic network map of the lemmas found in the dataset on social media (CrowdTangle) associated with “misinformation” and ”disinformation.” Node size represents lemma frequency, edges represent co-occurrence, and color and topology represent modularity class.

The entire retrieved dataset included 580 Facebook posts from pages and groups. From the analysis of the social media dataset obtained via CrowdTangle, the NLP analysis visualized through the co-occurrence analysis plot of the terms most commonly associated with “misinformation” and “disinformation” (Figure 3) and the semantic network map of the terms co-occurring in association with “misinformation” and disinformation” (Figure 4) reveals 4 relevant themes.

The first theme focuses on the proliferation of health-related misinformation during the pandemic, particularly on vaccinations and public health measures. Terms such as “Corona,” “Corona-Pandemie” (coronavirus pandemic), and “Corona-Impfung” (coronavirus vaccination), all present among the most commonly co-occurring terms with “misinformation” and “disinformation” (Figure 3), as well as the term “Durchseuchung” (herd immunity), highlight discussions on the pandemic’s progression and vaccine-related debates. Social media posts frequently questioned the effectiveness of vaccines, with claims often rooted in “Fehlinterpretation” (misinterpretation) or deliberate framing of scientific findings. Combined with terms such as “Schutz” (protection) and “Problem” (problem), this cluster seems to highlight a debate within public discourse around the protective benefits of vaccines and other interventions.

Ungeimpft. Eine Begegnung Eine Bekannte, die ich eigentlich recht gut mag, ist mit Ü50 noch nicht geimpft. Sie ist keine Massnahmen-Gegnerin und keine militante Schwurblerin. Sie hat einfach Angst vor möglichen Folgen einer Impfung. Auf die Frage, warum sie denn Angst habe, führt sie die ihr zugänglichen Informationen an. In den Medien seien ja immer wieder widersprüchliche Aussagen zu vernehmen. Studien und Gegenstudien, sogar Ärzte, die von der Impfung abraten. Meine Bekannte ist Künstlerin, Wissenschaft und Medien liegen ihr nicht nahe, haben in ihrem Leben selten eine Rolle gespielt. Jetzt sieht sie sich einem Wust aus Information ausgeliefert, die sie persönlich nicht einordnen kann. Sie ist überfordert. Sie anerkennt die Gefährlichkeit von Covid, und isoliert sich deshalb, trägt Maske, hält Distanz. Sie leidet unter ihrer Angst und auch an den Folgen ihrer Nicht-Impfung. Ihr Partner ist geimpft, sie streiten sich deswegen. Die Verantwortung für ihre Situation tragen Medien wie Nau, 20 Minuten oder die #SRFArena, die unwissenschaftlichen Scheissdreck gleichwertig neben wissenschaftlichen Erkenntnissen präsentieren, weil quoten- oder klickgeil. Meine Bekannte kann nicht zwischen einer MIT- oder ETH-Studie und einer Umfrage eines Füdli-Institutes unterscheiden, sie kann wissenschaftliche Forschung nicht einordnen, wie viele in unserer Gesellschaft. […] Und es ist Aufgabe des Staates, diese Informationen unzweifelhaft zu vermitteln. Auch in Schulen. Und sorry, da haben die meisten versagt. Wenn wir also Menschen haben, die sich aus Angst und Überforderung nicht impfen lassen, die sich isolieren, die klare Einordnung suchen, aber diese nicht bekommen, ist das ein gesellschaftliches Versagen. Die Medien haben den Schwurbler-Idioten eine breite Bühne geboten. Die Regierung hat sich vor den Massnahmen-Verweigerern eingepisst. Sorry, ihr habt versagt. Und jetzt geht, und räumt euren Scheiss auf.

Unvaccinated. An encounter. A friend of mine, who I actually like quite a lot, is over 50 and still not vaccinated. She is not opposed to the measures and is not a militant swearing person. She is simply afraid of the possible consequences of a vaccination. When asked why she is afraid, she cites the information available to her. After all, contradictory statements are repeatedly heard in the media. Studies and counter-studies, even doctors advising against vaccination. My acquaintance is an artist, science and the media are not close to her, have rarely played a role in her life. Now she finds herself at the mercy of a jumble of information that she personally cannot process. She is overwhelmed. She recognizes the danger of Covid, and therefore isolates herself, wears a mask, keeps her distance. She suffers from her fear and also from the consequences of not being vaccinated. Her partner is vaccinated, and they argue about it. The responsibility for her situation lies with media outlets like Nau, 20 Minuten or the #SRFArena, which present unscientific nonsense alongside scientific findings because they are hungry for ratings or clicks. My acquaintance cannot distinguish between a MIT or ETH study and a survey by a Füdli institute, she cannot classify scientific research, like many in our society. […] And it is the task of the state to communicate this information unequivocally. Also in schools. And sorry, most of them have failed there. So if we have people who, out of fear and overwhelm, don’t get vaccinated, isolate themselves, and seek clear categorization but don’t get it, that’s a social failure. The media has given the swearing idiots a broad stage. The government has pissed itself off the measure objectors. Sorry, you have failed. Now go and clean up your mess

[Row 13 CrowdTangle dataset, September 4, 2021]

Furthermore, public discourse within this theme included discussions on conspiracy theories, which dominated a significant portion of social media discourse, with terms such as “*QAnon”* (a global conspiracy theory movement), showcasing how global narratives infiltrated Swiss social media spaces. These theories often intersected with broader ideological narratives, such as antiestablishment sentiments and critiques of Western political structures (see the presence of the term “USA” in this corpus; Figure 4). Emotional terms, such as “Hetze” (hate speech) and “verkommen” (degraded), suggest the possible adversarial and polarized nature of these discussions, where extreme rhetoric (“extrem” is a term present in this cluster) fuels distrust. Posts associated with conspiracy theories frequently leveraged these narratives to legitimize alternative explanations and sow doubt about the government or the science behind the government’s decisions, showing how social media acted during the COVID-19 pandemic in Switzerland as a breeding ground for sensationalist and ideologically driven content.

Es bleibt nicht mehr viel Zeit, uns komplett dagegen zu wehren und uns für unsere demokratischen Grundrechte zu wehren! Sie wurden und werden gerade weltweit abgebaut! In der Schweiz unterlagen leider die Vernünftigen und das unterdrückerische Covidgesetz wurde wegen gezielter Angststreuung, und Desinformation durch den Bundesrat im Abstimmungsbüchlein, angenommen vom Souverän. Aber es geht nicht um ein Virus! Es geht um unsere Entrechtung und Enteignung! Die WHO soll neue WELTREGIERUNG im Pandemiefall werden! Und die können sie dank dafür nicht geeignetem PCR NAT jederzeit ausrufen! Mehrere Gerichtsurteile bestätigen, dass ein PCR NAT an Gesunden nichts aussagt! Auch sein Erfinder, der dafür einen Nobelpreis bekam, sagte das immer! Dr. Kari Mullis, USA, gestorben 2019.

There is not much time left to fight back completely and defend ourselves for our fundamental democratic rights! They have been and are being dismantled worldwide! In Switzerland, unfortunately, the sensible were subject to it and the oppressive Covid law was adopted by the sovereign because of the deliberate spreading of fear and disinformation by the Federal Council in the voting booklet. But it’s not about a virus! It’s about our disenfranchisement and expropriation! The WHO is supposed to become the new WORLD GOVERNMENT in the event of a pandemic! And thanks to the unsuitable PCR NAT, they can declare it at any time! Several court rulings confirm that a PCR NAT says nothing about the health of a person! Even its inventor, who received a Nobel Prize for it, always said that! Dr. Kari Mullis, USA, died in 2019

[Row 159 Crowdtangle dataset, April 20, 2022]

Äusserlich stimmt, dass die Summe aller Arbeitsplätze nicht dramatisch gesunken ist. Dies, weil die Staatsbürokratie auch in Corona-Zeiten – teils gerade wegen Corona – ungebremst ausgewuchert ist: Für all die akribische Kontrolle eingesperrter Bürgerinnen und Bürger sowie spitzfindiger Regulierung unterworfener Betriebe. Aber auch, weil weiterhin unverdrossen selbst für Unsinnigstes laufend neue Zweige der Funktionärsbürokratie neu geschaffen werden. In Zürich zum Beispiel eine «Fachkommission» zwecks Beratung des Stadtrats im Hinblick auf die Ausmerzung als rassistisch vermuteter Häuser-Namen. Gezielt wird dabei zum Beispiel auf das seit Jahrzehnten die Zwinglistadt schmückende «Haus zum Mohrenkopf».

On the surface, it is true that the total number of jobs has not fallen dramatically. This is because the state bureaucracy has continued to grow unchecked even in the age of the coronavirus – partly precisely because of the coronavirus: for all the meticulous control of locked-up citizens and the meticulous regulation of businesses. But also because new branches of the bureaucratic apparatus continue to be created unabated, even for the most nonsensical of reasons. In Zurich, for example, a “specialist commission” has been set up to advise the city council on the eradication of house names suspected of being racist. One of the targets is the “Haus zum Mohrenkopf,” which has graced the Zwinglistadt for decades

[Row 93 Crowdtangle dataset, March 18, 2022]

A second theme within the corpus explores the tension between public health measures and individual freedoms, framed through the lens of democracy and civil liberties. Terms such as “demokratisch” (democratic) and “Grundrecht” (fundamental right) reflect discussions about democratic values and protecting fundamental rights during crises. At the same time, terms such as “unterdrückerisch” (oppressive) and “souverän” (sovereign) suggest the presence of polarized narratives within social media discourse, where public health mandates were depicted as overreaching or infringing on personal rights and “sovereignty.” Further focusing on the polarized nature of social media discourse, the semantic network map (Figure 4) suggests that narratives of resistance and compliance clashed. Terms such as “wehren” (to resist) and “gezielt” (targeted), the latter present among the most co-occurring terms with “misinformation” and “disinformation” (Figure 3), suggest organized resistance against perceived institutional overreach, with “abbauen” (dismantle) possibly reflecting arguments aimed at dismantling or modifying public health measures, whereas on the contrary, discussions also framed mandates as reasonable (“vernünftigen”). For example:

«Der Bundesrat entschädigt den Schaden, den er selber angerichtet hat nur, wenn er Vollmachten bekommt. Das ist Erpressung.» Nicolas A. Rimoldi, Co-Präsident von MASS-VOLL!, debattiert gegen Manuela Weichelt, Nationalrätin der Grünen, zum Covid-19-Gesetz. Uns wird seit mehr als einem Jahr eine alternativlose Politik präsentiert, die suggeriert, dass nur das massive Eingreifen des Staates in allen Bereichen des Lebens einen Weg aus der selbstverschuldeten Krise darstellt. Dass die vom Bundesrat getroffenen Massnahmen die wirtschaftliche, soziale und politische Krise verursacht haben, wird ignoriert. Die im Covid-19-Gesetz geregelten Finanzhilfen sollen den geschädigten Unternehmen Abhilfe schaffen – doch ein Gesetz, welches die Souveränität des Volkes massiv einschränkt und eine Zweiklassengesellschaft einführt, darf in unserer Demokratie keine ernsthafte Option sein. Sowieso: Die “Entschädigungen” sind nur Almosen, sie helfen nicht. Nur ein NEIN führt dazu, dass alle vollumfassend entschädigt werden! Nutze Deine Stimme für ein NEIN zum Covid-19-Gesetz am 13. Juni - ein NEIN zu Entmachtung, zu Willkür und zu einer alternativlosen Politik. Unterstütze auch Du MASS-VOLL! und sag JA zur Selbstbestimmung. ✊🏼💜Quelle: Kontrovers – Zentralschweizer Fernsehen

Manuela Weichelt, member of the National Council for the Greens, on the Covid-19 Act. For more than a year, we have been presented with a policy with no alternative, suggesting that the only way out of the self-inflicted crisis is massive state intervention in all areas of life. The fact that the measures taken by the Federal Council have caused the economic, social and political crisis is being ignored. The financial aid regulated by the Covid-19 Act is intended to provide relief for the companies that have suffered damage - but a law that massively restricts the sovereignty of the people and introduces a two-class society must not be a serious option in our democracy. In any case: the “compensation” is just charity, it does not help. Only a NO vote will lead to everyone being fully compensated! Use your vote for a NO to the Covid-19 law on June 13—a NO to disempowerment, to despotism and to a policy without alternatives. You too can support MASS-VOLL! and say YES to self-determination. ✊💜 Source: Kontrovers - Zentralschweizer Fernsehen

[Row 6579 Crowdtangle Dataset, May 29, 2021]

The third theme captured the phenomenon of information overload during the pandemic, where the volume of information—both accurate and false—left the public feeling “überfordert” (overwhelmed), a term strongly associated with “misinformation” and “disinformation” in our analysis (Figure 3). Terms such as “Statistik” (statistics) and “falsch” (false), also among the most commonly co-occurring terms with “misinformation” or “disinformation” (Figure 3), point to discourses questioning the accuracy and reliability of reported data on COVID-19 cases, deaths, and hospitalizations. In addition, terms such as “psychisch” (psychological) and “Störung” (disorder) likely reflect growing concerns about mental health issues linked to prolonged restrictions (“Einschränkung”) and the fear-driven narratives spread online. The term “*beispiellos”* (unprecedented) highlights the “unprecedented” nature of these challenges, while “Hospitalisierungen” (hospitalizations) suggests anxiety around the strain on health care systems. All the abovementioned terms, from “psychisch” to “Hospitalisierungen,” are included in the list of the most commonly co-occurring terms with “misinformation” and “disinformation” (Figure 3). Furthermore, the presence of the term “Demokratie” (democracy) within this thematic cluster in the semantic network map (Figure 4) reveals that the discourse on fear and anxiety exacerbated by misinformation and disinformation touched upon the tensions and balance between maintaining democratic norms and implementing effective public health measures.

The fourth theme identified suggests that social media discourse in Switzerland was shaped around the relationship between misinformation and disinformation and democratic processes. Terms such as “Demokratie” (democracy) and “*unabhängig”* (independent) point to concerns within public discourse about the erosion of democratic norms due to the spread of misinformation and disinformation, with narratives often calling for accountability (“aufdecken”). The centrality of the term “*mensch”* (human, person) in this cluster—featured as a central node in the semantic network map (Figure 4) and prominently listed among the terms most frequently co-occurring with “misinformation” and “disinformation” (Figure 3)—underlines the human impact of misinformation and disinformation, with discussions focusing on how misinformation and disinformation affects individuals and communities. The term “Verantwortung” (responsibility) likely reflects calls for collective accountability, with an emphasis on the ethical obligation to counteract narratives that are harmful (“schädlich”). Terms such as “Bewegung” (movement) and “verteidigen” (to defend) possibly suggest grassroots efforts within social media discourse to defend what they perceive as human values and societal norms. This theme suggests that, beyond institutional, ideological, and polarized debates, the ultimate stakes of misinformation and disinformation are their tangible impacts on human lives and social cohesion, as recognized by people shaping public discourse on social media.

Als ich die Nachricht hörte, dachte ich an einen schlechten Scherz. Doch das Bundesamt für Gesundheit (BAG) bestätigt entsprechende Pressemeldungen: Die Beamten im Innendepartement von SP-Bundesrat Alain Berset haben heimlich einen sogenannten «Fake-News-Monitor» eingerichtet. «Eine spezielle Software wertet für die Behörden soziale Medien, Nachrichtenportale und Zeitungen nach (Falsch-)Informationen aus. So erhalten sie täglich einen Überblick über die Informationen, die in der Schweiz kursieren», schreibt der «Blick». Und fügt treuherzig hinzu, die Software diene dem BAG als «Frühwarnsystem». So könne der Bund mit «Kampagnen» auf Desinformation reagieren, etwa zum Thema Impfen. Ein «Wahrheitsministerium» wie bei Orwell Ich weiss nicht, wie es Ihnen geht – aber mir läuft es dabei kalt den Rücken herunter. Ein Bundesamt für Staatspropaganda? Ein «Wahrheitsministerium»? Irgendwie kommt einem das bekannt vor. George Orwell hat dies in seinem antitotalitären Zukunftsroman «1984», geschrieben 1948, anschaulich vorweggenommen.

When I heard the news, I thought it was a bad joke. But the Federal Office of Public Health (FOPH) confirmed the corresponding press reports: the officials in the interior department of SP Federal Councillor Alain Berset have secretly set up a so-called “fake news monitor.” “Special software evaluates social media, news portals and newspapers for (false) information for the authorities. This provides them with a daily overview of the information circulating in Switzerland,” writes the Blick. And adds, ingenuously, that the software serves as an ‘early warning system’ for the FOPH. This would enable the federal government to respond to disinformation with “campaigns,” for example on the subject of vaccination. A “Ministry of Truth” like in Orwell I don’t know about you, but it sends a shiver down my spine. A federal office for state propaganda? A “Ministry of Truth”? Somehow it seems familiar.

[Row 59 Crowdtangle dataset, 22.08.2021]

## Discussion

### Contested Meanings of Misinformation and Disinformation in Swiss Public Discourse

The analysis shows that the understanding, conceptualization, and use of “misinformation” and “disinformation” operate as a critical point of contention, balancing the priorities of individual liberties and collective responsibilities during times of crisis. This dynamic reflects the inherent tensions in public discourse, as debates clash with the trade-offs between personal freedoms and the broader public good. This aligns with previous findings on Swiss public discourse, where the concept of “freedom” was shaped within the broader debate on balancing individual and collective rights [20]. News media focused on evaluating the impacts of misinformation and disinformation, particularly the tension between journalistic integrity and the amplification of falsehoods. This occurred within a fragmented and polarized information environment, in which social media platforms provided a fertile ground for polarized narratives [34-36]. These narratives frequently intersected with conspiracy theories, emotional appeals, and ideological conflicts, exacerbating societal polarization [37]. If the Factiva dataset captured a narrative of journalistic accountability, highlighting journalism’s role in mitigating the spread of falsehoods, the CrowdTangle dataset revealed a markedly different pattern, dominated by a discourse characterized by emotionally charged content and organized resistance to perceived institutional overreach. In mainstream media, misinformation and disinformation are generally considered a challenge, with debates about editorial responsibility and the role of fact-checking in debunking misinformation and disinformation. By contrast, on social media, the same concepts are often discussed and interpreted, as we have seen, as instruments of control, with fact-checking seen as a threat to freedom of expression. We argue that this divergence illustrates a fracture between institutional and grassroots narratives, causing a fragmented and polarized public discourse, where efforts to preserve informational accuracy in one arena can be perceived as censorship in another. We argue that this reflects competing epistemic frameworks that can undermine the existence of points of contact between diverging views and opinions in Swiss society, causing damage and fueling polarization.

In light of these findings and the ongoing societal debates about fact-checking and content moderation [38], we explore potential strategies to reduce fragmentation within polarized public discourse, recognizing that addressing this challenge requires a careful balance between promoting open debate and mitigating the influence of misinformation and disinformation. Rather than assuming that public conversations and differing opinions naturally emerge from well-informed deliberation, we acknowledge that many individuals engage with information in ways that are shaped by cognitive biases, social dynamics, and varying levels of health and media literacy [39]. Moreover, the quality and reliability of the information on which people base their choices are not only essential for individual decision-making but also a prerequisite for democracies that are not merely formal but substantive—where civic participation is grounded in informed judgment rather than manipulation or epistemic asymmetries. Thus, any effort to strengthen public discourse must consider the complexity of how information is processed and acted upon.

For news media, we consider the role of fact-checking mechanisms and editorial accountability as essential tools for ensuring the reliability of information, which need systematic application. While robust verification processes can help reduce the spread of false or misleading content, their effectiveness depends on how deeply they are integrated into journalistic culture and practices, and whether audiences trust and engage with them. Additionally, editorial standards should not only prevent the dissemination of inaccuracies but also encourage nuanced reporting that resists sensationalism and oversimplification, which can contribute to polarization even in the absence of outright falsehoods.

For social media platforms, initiatives aimed at enhancing public engagement and improving information literacy have potential benefits. While some interventions—such as algorithmic adjustments to prioritize credible sources—may help reduce exposure to misinformation, they must be implemented with caution to avoid unintended consequences, such as reinforcing ideological silos, echo chambers, or diminishing pluralism. Likewise, efforts to promote information literacy should not be framed solely as a reactive defense against manipulation, but as a broader proactive means of cultivating critical thinking skills that enable individuals to assess claims.

The extent to which these approaches can mitigate the spread of misinformation and enhance public resilience to its effects likely depends on multiple factors, including institutional cooperation, societal trust in information sources, and the willingness of individuals to engage in reflective, rather than reactive, forms of reasoning. In this sense, this study contributes to understanding societal resilience not merely as an outcome of effective crisis communication, but as a process sustained by epistemic robustness, trust, and deliberative inclusiveness—arguably, resilience is rooted in the capacity of societies to maintain transparent, plural, and reasoned public debate even under conditions of uncertainty. Acknowledging these challenges, we argue, promotes an epistemic culture in which knowledge is critically examined, rather than passively received or reflexively accepted or rejected; this may be a more sustainable long-term goal than attempting to eliminate misinformation outright.

### Limitations

This study has certain limitations that should be considered when interpreting the findings. First, the representativeness of the datasets is limited by design: social media data, collected via CrowdTangle, capture publicly shared content, potentially overrepresenting perspectives from certain demographic groups while underrepresenting others, such as individuals who prefer private interactions or alternative communication platforms. Second, although the datasets enable longitudinal analysis, this study does not systematically examine temporal variations in discourse. Third, while the findings highlight significant trends and divergences in framing misinformation and disinformation, the analysis necessarily simplifies complex social constructs, possibly without fully capturing nuances and flattening the complexity of public discourse. Finally, for future research, there is a need for a deeper, qualitative examination of how misinformation and disinformation have been addressed in public discourse during the COVID-19 pandemic.

## Abbreviations

NLP: natural language processing
